# Hepatitis C VLPs Delivered to Dendritic Cells by a TLR2 Targeting Lipopeptide Results in Enhanced Antibody and Cell-Mediated Responses

**DOI:** 10.1371/journal.pone.0047492

**Published:** 2012-10-16

**Authors:** Brendon Y. Chua, Douglas Johnson, Amabel Tan, Linda Earnest-Silveira, Toshiki Sekiya, Ruth Chin, Joseph Torresi, David C. Jackson

**Affiliations:** 1 Department of Microbiology and Immunology, The University of Melbourne, Parkville, Victoria, Australia; 2 Department of Infectious Diseases, Austin Hospital, Heidelberg, Victoria, Australia; 3 Department of Medicine, Austin Hospital, The University of Melbourne, Heidelberg, Victoria, Australia; 4 Department of Medicine, Austin Hospital, The University of Melbourne, Heidelberg, Victoria, Australia; St.Louis University, United States of America

## Abstract

Although many studies provide strong evidence supporting the development of HCV virus-like particle (VLP)-based vaccines, the fact that heterologous viral vectors and/or multiple dosing regimes are required to induce protective immunity indicates that it is necessary to improve their immunogenicity. In this study, we have evaluated the use of an anionic self-adjuvanting lipopeptide containing the TLR2 agonist Pam_2_Cys (E_8_Pam_2_Cys) to enhance the immunogenicity of VLPs containing the HCV structural proteins (core, E1 and E2) of genotype 1a. While co-formulation of this lipopeptide with VLPs only resulted in marginal improvements in dendritic cell (DC) uptake, its ability to concomitantly induce DC maturation at very small doses is a feature not observed using VLPs alone or in the presence of an aluminium hydroxide-based adjuvant (Alum). Dramatically improved VLP and E2-specific antibody responses were observed in VLP+E_8_Pam_2_Cys vaccinated mice where up to 3 doses of non-adjuvanted or traditionally alum-adjuvanted VLPs was required to match the antibody titres obtained with a single dose of VLPs formulated with this lipopeptide. This result also correlated with significantly higher numbers of specific antibody secreting cells that was detected in the spleens of VLP+E_8_Pam_2_Cys vaccinated mice and greater ability of sera from these mice to neutralise the binding and uptake of VLPs by Huh7 cells. Moreover, vaccination of HLA-A2 transgenic mice with this formulation also induced better VLP-specific IFN-γ-mediated responses compared to non-adjuvanted VLPs but comparable levels to that achieved when coadministered with complete freund’s adjuvant. These results suggest overall that the immunogenicity of HCV VLPs can be significantly improved by the addition of this novel adjuvant by targeting their delivery to DCs and could therefore constitute a viable vaccine strategy for the treatment of HCV.

## Introduction

Hepatitis C virus (HCV) infection affects an estimated 200 million individuals worldwide and contributes to significant morbidity and mortality rates associated with liver cirrhosis and hepatocellular carcinoma. Approximately 80% of infected individuals do not clear the virus following acute infection and will develop chronic infection that can lead to end-stage liver disease and complications. Although treatment options using a combination of pegylated interferon-α and ribavirin are available, sustained clearance of the virus is only achieved in approximately 40% of individuals infected with HCV genotype 1 and 60–70% of those who are infected with genotypes 2 or 3 [1]. Recent advances in the treatment of HCV using directly acting antiviral agents (DAAs) such as boceprevir and telaprevir have improved SVR rates in both treatment naïve and experienced patients (reviewed in [2]). However, treatment can be prolonged, expensive and also associated with substantial side effects. The development of an effective vaccine that can significantly reduce the number of new infections and improve sustained virological response rates could therefore be a useful adjunct to current therapeutic approaches and reduce the impact of infection on global health care systems.

Whilst the immune correlates mediating the clearance of virus are still not entirely clear or defined, there is substantial evidence demonstrating that the development of a broad multifunctional T cell response against an array of key viral proteins such as core, E1, NS3, NS4 and NS5 during acute HCV infection is associated with disease resolution [3], [4] and may also provide a level of protection against reinfection [5]. It is also becoming increasingly apparent that such responses alone are not enough [6] and that neutralising antibodies also play an integral role in conferring protection [7], [8] and facilitating viral clearance by mechanisms including antibody-dependent cellular cytotoxic mechanisms [9]. An effective HCV vaccine will need to induce antibody and cell-mediated responses and also provide cross protection against different viral genotypes and quasispecies. Neutralising antibodies induced against conserved, conformational epitopes in the viral envelope E1 and E2 glycoproteins [10]–[12], notably antigenic region 3 (AR3)[13] of E2, including the critical neutralisation contact residues contained within domain I of E2 [14] and amino acids 313–327 of E1 [15], can be broadly cross-neutralising. The fact that these antibodies neutralise different HCV genotypes highlights the importance of including epitopes from both envelope proteins for a vaccine strategy to be effective.

Virus-like particles (VLPs) possess features which make them ideal vehicles for the delivery of viral antigens to the immune system; (i) antibody epitopes are presented in the native conformation for induction of potentially neutralising antibodies (ii) multiple T cell, CD4^+^ and CD8^+^, epitopes are packaged in VLPs (iii) VLPs lack regulatory proteins as well as genetic material that could pose a risk of reversion or mutation (iv) encouraging results have been obtained using insect cell-derived recombinant VLPs expressing HCV antigens which induce virus-specific humoral and cellular responses [16]–[18] (v) HCV VLPs appear to possess properties favourable for dendritic cell uptake [19] and (vi) they exhibit superior immunogenicity and antigenicity over recombinant protein and DNA-based vaccine approaches [16], [17].

An important consideration in the manufacture of HCV-based VLPs is the cell-type used for their manufacture. For example, it has been shown that vaccination with recombinant HCV envelope proteins expressed in mammalian cells, but not in yeast or insect cells, protect chimpanzees from primary infection by an homologous HCV isolate [20]. Similarly, Rosa *et al* have demonstrated that mammalian cell-derived recombinant envelope proteins bind to human cells with higher affinity than those produced in yeast or insect cells and appear to be antigenically and functionally similar to the viral proteins produced in an infected host cell [21]. More recently, vaccination of macaques using VLPs in a prime-boost regime has been reported to induce broadly neutralising antibody responses against different HCV genotypes [22]. Although all of these studies provide encouraging results supporting the development of HCV VLP-based vaccines, the fact that heterologous viral vectors and/or unrealistic dosing regimes are required to induce protective immunity indicates that it is necessary to improve their immunogenicity.

In this study we evaluate the immunogenicity of mammalian cell-derived VLPs containing structural proteins (core, E1 and E2) of HCV genotype 1a when delivered directly to dendritic cells (DCs) using a Toll-like receptor 2 (TLR2) targeting lipopeptide. This lipopeptide contains the TLR2 agonist dipalmitoyl-S-glyceryl-cysteine (Pam_2_Cys) and associates electrostatically with protein antigens significantly improving their ability to induce both humoral and cell-mediated responses [23]. The ability of lipopeptide-VLP complexes to facilitate DC uptake, induce antibody capable of inhibiting VLP entry into target cells and to elicit cell-mediated antigen-specific responses were each determined.

## Materials and Methods

### Assembly of HCV VLPs

HCV virus-like particles were constructed using a recombinant adenovirus containing encoding the HCV structural proteins (core, E1 and E2) of HCV 77H, genotype 1a. Briefly, the core/E1 genes were amplified from pBRTM_HCV 1–3011 plasmid containing the genome of HCV H77 genotype 1a (a gift from Prof C Rice). The core/E1 genes were amplified from pBRTM_HCV 1–3011 plasmid containing the genome of HCV H77 genotype 1a (a gift from Prof C Rice). The forward Core/E1 primer (**5′**gCCTCTAgAgCCACCATgCATCACCATCACCATCACACAAgCACgAATCCTAAACTCAAAgAAAAACC **3′**) was designed to introduce an *Xba*I enzyme restriction site followed by a Kozak sequence, a start codon and a His(6) tag at amino terminal end of the core protein. The reverse primer of core/E1 (**5′** ggCTTAAgCCCggTgACgTgggTTTCC gCgTCgAC **3′**) was designed to amplify from the sequence downstream of the region corresponding to E1/E2 cleavage site (amino acids 383/384) and to introduce an *Afl* II restriction site at the 3′ end. Next, the E2 genome was amplified using a forward primer (**5′**CgACTAgTgAAACCCACgTCACCgggggAAgTgCCggCCgC **3′**) that also introduced a *Spe*I site at the 5′ end. The reverse E2 primer (**5′**CgGATATCTCATCAC gCCTCCgCTTgggATATgAgTAACATCATCC **3′**) was designed to introduce a double stop codon and an *Eco*RV restriction site at the 3′ end of the amplicon.

The core/E1 and E2 amplicons were cloned into pGEMEasy (Promega) and subsequently subcloned and ligated to produce a construct which was verified by DNA sequencing. This construct was subsequently subcloned into pAdTrack-CMV (provided by B Vogelstein, Howard Hughes Medical Centre, Baltimore), digested with PmeI and transformed into AdEasier-1 cells by electroporation (Bio-Rad Gene Pulser) as previously described [24].

### Production and Purification of HCV VLPs

High titres of recombinant adenovirions encoding the HCV proteins (rAdHCV-CE1E2) were produced in 293T cells by serial passaging and the equivalent multiplicity of infection (MOI) was determined as described previously [24]. To produce HCV VLPs, Huh7 cells were infected with rAdHCV-CE1E2 at a MOI of 1. At 72 hours post-infection, cells were collected and disrupted using a dounce homogeniser and centrifuged at 17,000 g for 5 min. The supernatant was further centrifuged through a 30% sucrose cushion (containing 20 mM Tris pH 7.4 and 150 mM NaCl) at 178,000 g for 4 hours at 4°C. The resulting pellet was resuspended in 50 mM Tris pH 7.and 100 mM NaCl and purified through a 33% caesium chloride gradient by ultracentrifugation at 14°C at 143,000 g) for 72 hours. Twelve 1 ml gradients were recovered and dialysed against sterile PBS at 4°C overnight. Fluorescent labelling of VLPs was achieved by adding VLPs (6 mg/ml) to 2 mg/ml of fluorescein isothiocyanate (FITC; Sigma Aldrich) in 50 µl of DMSO. The suspension was vortexed vigorously and incubated overnight at 4°C before dialysis against PBS the next day. All VLP preparations were stored in aliquots at −70°C until use. Huh7 and 293T cells were grown in Dulbecco’s modified Eagle’s medium (DMEM; Invitrogen USA) supplemented with 10% fetal calf serum (FCS) and streptomycin 50 µg/ml at 37 C in 5% CO_2_.

### Synthesis of Anionic Lipopeptide E_8_(Pam_2_Cys)

**Figure 1:**
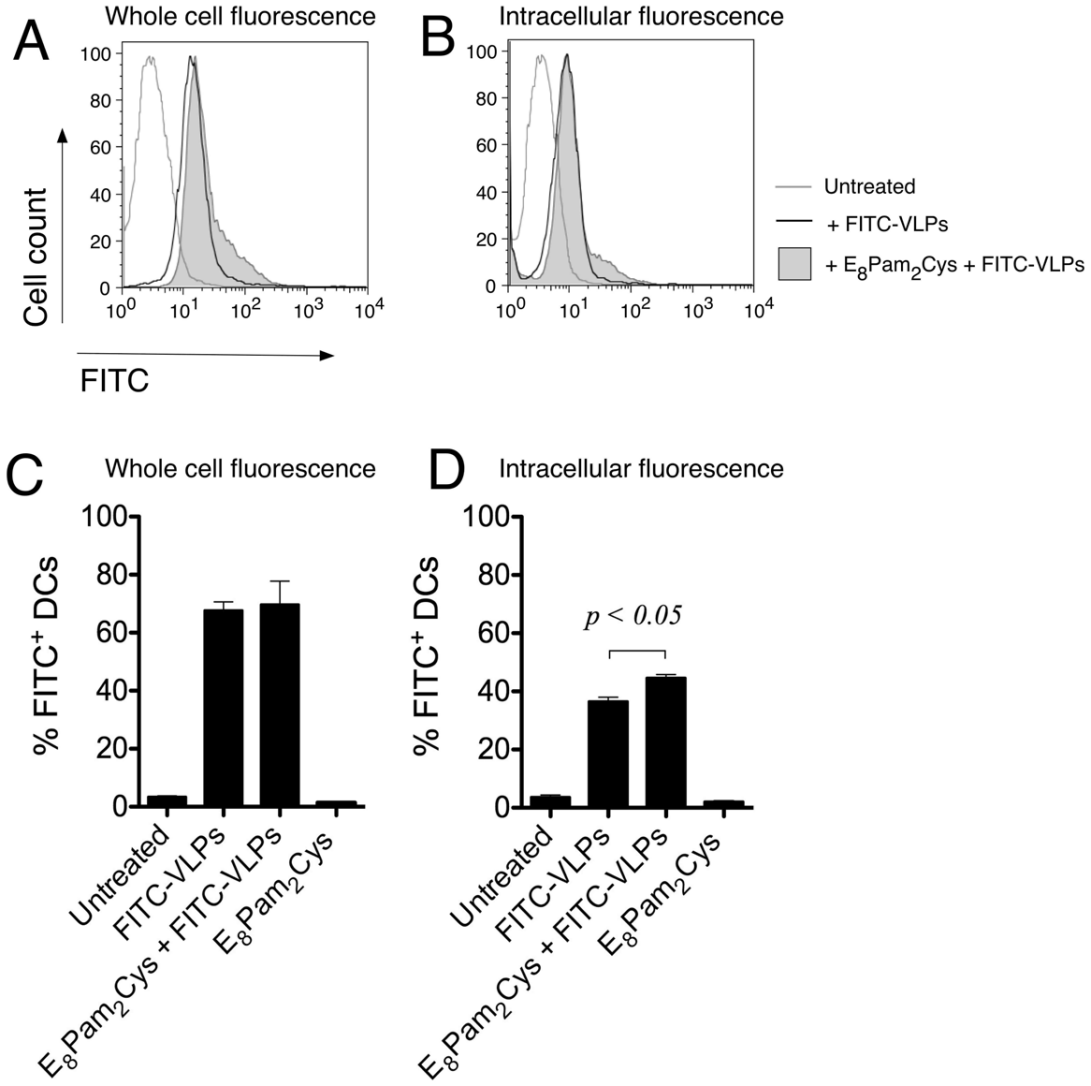
Dendritic cell uptake of fluorescenated VLPs. CD11c^+^ MHC Class II^+^ splenocyte-derived cultured D1 dendritic cells (2×10^5^ cells) were cultured in the presence of 5 µg FITC-labelled VLPs alone or in association with E_8_Pam_2_Cys (0.01 nmoles/ml). Cells were harvested after 24 hours, washed, fixed in 1% paraformaldehyde and analysed for green fluorescence by flow cytometry. Representative histograms show both surface (A) and intracellular (B) associated fluorescence derived from the analysis of a minimum of 1×10^4^ cells in each sample. To determine the level of fluorescence emanating from the cell interior, extracellular fluorescence was quenched by exposing cells to trypan blue for 1 minute prior to analysis. Bar graphs show the percentage of cells deemed to be positive for (C) whole cell or (D) intracellular associated fluorescence based on a marker that was set using untreated cells and are representative of results from one of three experiments conducted separately.

The syntheses of the branched anionic peptide construct containing eight N-terminal glutamic acid residues (E_8_) using traditional Fmoc chemistry has been described previously [23]. Briefly, synthesis was carried out manually using PEG-S RAM solid support (Rapp Polymere, Tübingen, Germany; substitution factor 0.27 mmol/g). Fmoc-lysine(Mtt)-OH (Novabiochem, Läufelfingen, Switzerland) was first coupled to the support and the Fmoc protecting group present on the α-amino group then removed and Fmoc-lysine(Fmoc)-OH was then coupled to the exposed N-terminal amino group. Subsequent de-protection and acylation of another two rounds of Fmoc-lysine(Fmoc)-OH yielded eight branch points to which glutamic acid residues were coupled. The primary amino groups of the glutamic acid residues were then acetylated using a 20-fold excess of acetic anhydride and a 40-fold excess of diisopropylethylamine (DIPEA; Sigma, Australia) to generate E_8_ which has an overall charge of −8. Lipidation of E_8_ was then carried out by removing the Mtt protective group present on the ε-amino group of the C-terminal lysine followed by acylation of the exposed ε-amino group with two serially added serine residues. The Pam_2_Cys lipid moiety was then coupled according to Zeng et al [25] to generate E_8_(Pam_2_Cys) (Figure 1A).

Following assembly, lipopeptides were cleaved from the solid phase support and all side-chain protecting groups removed with 88% TFA, 5% phenol, 2% TIPS, 5% water for 3 hours at room temperature. Lipopeptides were analysed by reversed phase high-pressure liquid chromatography (RP-HPLC) using a Vydac C4 column (4.6×300 mm) installed in a Waters HPLC system. The chromatogram was developed at a flow rate of 1 ml/min using 0.1% TFA in H_2_O and 0.1% TFA in acetonitrile as the limit solvent. Lipopeptides were purified if necessary. All products presented as a single major peak on analytical RP-HPLC and had the expected mass when analysed using an Agilent series 1100 ion trap mass spectrometer.

### Uptake of VLPs and Maturation of DCs

A line of murine BALB/c-derived DCs (D1 cells) was prepared and propagated according to the method described by Chua et al [26]. After a minimum of 21 days in culture, cells were stained for Class II MHC using FITC conjugated anti-IA/IE antibody (Clone M5/114.15.2; Becton Dickinson, USA) and PE-conjugated CD11c (Clone 2G9; Becton Dickinson, USA) prior to use. Cells were verified to be CD11c^+^MHC Class II^+^ by flow cytometry using a FACSCaliber (Becton Dickinson, USA). D1 cells (2×10^5^) were seeded onto a petri dish in 1 ml of fresh D1 media [26] and incubated at 37°C and 5% CO_2_ in the presence or absence of 5 µg FITC-labelled VLPs alone or with VLPs mixed with E_8_Pam_2_Cys (0.2 pmole/ml).

Cells were harvested 24 hours later and washed with FACs wash (1% FCS/5 mM EDTA in PBS) before fixation in 1% paraformaldehyde in PBS. To examine cellular association of VLPs, cell fluorescence was analysed by flow cytometry (FACSCaliber, Becton Dickinson, USA). For examination of intracellular uptake of VLPs, extracellular fluorescence was quenched by addition of an equal volume of 0.1 M citrate buffer (pH 4.0) containing 250 µg/ml trypan blue (Merck, Damstadt, Germany) prior to analysis [27]. Data were analysed using FlowJo software (Tree Star, San Carlos, CA).

To assess the degree of DC maturation resulting from exposure to different adjuvants, cells were exposed to varying concentrations of aluminium hydroxide gel, Alhydrogel (Sigma Aldrich, Missouri, USA), E_8_Pam_2_Cys or lipopolysaccharide (5 µg/ml) as a positive control (LPS; Sigma Aldrich, Milwaukee, USA). In some experiments, cells were incubated with VLPs alone (5 µg/ml) or in the presence of E_8_Pam_2_Cys (0.032 µg/ml). After 16 hours, cells were harvested, washed and analysed for expression of surface Class II MHC antigen.

### Detection of IFN-γ-secreting Cells by ELISPOT

All experimental procedures involving animals were approved by the University of Melbourne’s animal ethics committee under the AEC numbers 0707207 and 061061. HLA-A2k^b^ transgenic mice (HHD mice) were obtained from the Queensland Institute for Medical Research and bred in the Animal House Facility of the Department of Microbiology and Immunology at The University of Melbourne under specific pathogen free conditions. These mice do not express H-2D^b^ but instead express the chimeric monochain of the α1 & α2 domains of HLA-A2.1 and the α3 cytoplasmic and transmembrane domains of H-2D^b^ linked at its N-terminus to the C terminus of human β2 microglobulin [28].

Female HHD mice (3 per group) were inoculated subcutaneously on each side of the base of tail (50 µl per dose) with VLPs (30 µg) either alone, emulsified in an equal volume of complete Freund’s adjuvant (CFA) or with an equal amount of E_8_Pam_2_Cys on days 0 and 14. Spleens were removed 28 days after the second dose and splenocytes restimulated *in vitro* at a concentration of 3×10^6^ cells/ml in RF-10 medium consisting of RPMI 1640 medium (Gibco, USA) supplemented with 10% fetal calf serum (CSL, Parkville, Australia) 7.5 mM HEPES, 2 mM L-glutamine, 76 µM 2-mercaptoethanol, 150 U/ml penicillin, 150 µg/ml streptomycin, 150 µM non-essential amino acids and 10 U/ml of recombinant IL-2 (Roche, Indianapolis, USA) at 37°C in an atmosphere of 5% CO_2_. Restimulation was carried out in the presence of 10 µg of VLPs or 10 µM of an irrelevant HCV-derived HLA-A2-restricted epitope (NS5B2594–2602) that is not contained in the VLP construct.

Cells were harvested 5 days later, washed and serial dilutions commencing at 5×10^5^ cells/ml performed in polyvinylidene fluoride (PVDF) membrane-lined 96-well plates (Millipore, Ireland) previously coated with 5 µg/ml anti-IFN-γ capture antibody (clone R4-6A2-BD Pharmingen, San Diego, USA). Cells were then cultured in the presence of 3.75×10^5^ irradiated autologous VLP-pulsed (10 µg) splenocytes for 40 hours at 37°C and 5% CO_2_. After washing with PBST (PBS containing 0.05% Tween 20), biotinylated anti-IFN-γ detection antibody (clone XMG1.2; Becton Dickinson, USA) was added and incubated for 2 hours at room temperature in a humidified atmosphere. Plates were then washed and streptavidin-alkaline phosphatase (Becton Dickinson, USA) added and incubated for a further 2 hours. Spots representative of IFN-γ-producing cells were developed by the addition of 100 µl of 1 mg/ml 5-bromo-4-chloro-3-indolyl phosphate in 2-amino-2-methyl-1-propanol buffer (Sigma-Aldrich, USA) for 30 minutes. Individual spots were counted using an AID iSpot EliSpot Reader (GmbH, Strassberg, Germany). Analysis of variance in all experiments and all *p* values in this study were conducted and obtained using one-way ANOVA nonparametric statistical analysis and Tukey’s post-hoc range tests performed with Prism 5 (GraphPad Software, La Jolla, California USA).

### Measurement of Specific Antibody Production by ELISA

Female 6–8 week old BALB/c mice were inoculated subcutaneously on each side at the base of the tail (50 µl per dose) on days 0 and 21 with either VLPs (20 µg) alone, VLPs pre-incubated with an equal volume of Alhydrogel (13 mg/ml) or 60µg of E_8_Pam_2_Cys. An additional dose of each formulation was administered to 3 mice from each group on day 56. Sera were prepared from blood taken on days 21, 35 and 63.

Flat bottom 96-well polyvinyl plates were coated with either purified HCV VLPs (10 µg/ml) or recombinant E2 protein (5****µg/ml) in PBSN_3_ overnight at 4°C. Prior to coating plates with HCV VLPs, wells were pre-incubated with Galanthus nivalis lectin (2 µg/ml; Sigma Aldrich Australia) in carbonate buffer (15 mM NaCO_3_, 35 mM NaHCO_3_, 0.21 mM NaCl) for 30 minutes at room temperature Following removal of antigen, 100 µl of BSA (10 mg/ml) in PBS was added and plates incubated for 1 hour at room temperature before washing four times with PBST (PBS containing v/v 0.05% Tween-20 [Sigma Aldrich, Milwaukee, USA]). Serial dilutions of sera obtained from immunised mice were added to wells and incubated in a humidified atmosphere overnight. After washing, bound antibody was detected using horseradish peroxidase-conjugated rabbit anti-mouse IgG antibodies (Dako, Glostrup, Denmark) in conjunction with enzyme substrate (0.2 mM 2,2_-azino-bis 3-ethylbenzthiazoline-sulfonic acid in 50 mM citric acid containing 0.004% hydrogen peroxide). The reaction was stopped by addition of 50 µl of 0.05 M NaF. Titers of antibody are expressed as the reciprocal of the highest dilution of serum required to achieve an optical density of 0.2.

### Measurement of B Cell Specific Antibody Production by ELISPOT

For the detection of specific antibody secreting cells by ELISPOT, PVDF membrane-lined 96-well plates (Mabtech, Nacka Strand, Sweden) were coated with 100 µl of PBS containing VLPs (10 µg/ml), recombinant E2 (10 µg/ml) or anti IgG antibody (10 µg/ml) overnight at 4°C. Plates were washed 5 times with PBS and blocked for 2 hours using RPMI 1640 medium (Gibco, USA) supplemented with 20% BSA (Sigma, Australia). Wells were emptied before 5×10^5^ splenocytes in 200 µl of RF-10 medium was added and incubated for 36 hours at 37°C in an atmosphere of 5% CO_2_. Spot forming units representative of specific antibody-producing cells were developed as previously described for the detection of IFN-γ-secreting cells except that biotinylated anti-IgG antibody and streptavidin-conjugated horse-radish peroxidase (both from Mabtech, Nacka Strand, Sweden) were used as detecting reagents.

### HCV Neutralisation Assay

In order to determine any inhibition of cell entry by VLPs using antibodies present in sera of vaccinated animals, Huh7 cells were first incubated with PBS (10% FCS) for 15 min at 4°C to reduce non-specific binding of antibodies subsequently added. Cells were washed twice, resuspended in PBS (0.1% FCS) and incubated with FITC-labelled VLPs at 4°C for 1 hr. Serial dilutions of sera from vaccinated or non-vaccinated mice were then added and incubated for a further 1 hour at 37°C. For each reaction, 5×10^5^ Huh7 cells and 200 ng of FITC-labelled VLPs were used in a total volume of 500 µl. At the end of this incubation period, cells were washed with PBS (0.1% FCS) and then fixed in BD Cytofix (Becton Dickinson, USA). Inhibition of VLP entry was determined by flow cytometry and analysed using WEASEL 2.0 software (Walter and Eliza Hall Institute, Melbourne, Australia). Sera from vaccinated mice that demonstrated a decrease in specific cellular binding of 50% or more compared to sera from naïve mice were considered to contain neutralising antibodies [18].

## Results

### Dendritic Cell VLP Uptake and Maturation

HCV genotype 1a VLPs were produced by transducing a human hepatocyte-derived cell line with recombinant adenovirus containing encoding the HCV structural proteins (core, E1 and E2) of genotype 1a to produce particles that harbour antigenic resemblance to virions produced in an infected host cell. Because of the essential role that DCs play in the induction of both humoral and cell-mediated responses, we first examined the ability of a spleen-derived DC line (D1 cells) to take up fluorescein isothiocyanate-labelled HCV VLPs (FITC-VLPs). Flow cytometric analysis revealed that DCs incubated with FITC-VLPs exhibited higher whole cell fluorescence intensities compared to untreated DCs indicating the presence of cell-associated VLPs (Figure 1B). Exposure of DCs to VLPs pre-mixed with the lipopeptide E_8_Pam_2_Cys also resulted in higher levels of cell fluorescence compared to untreated DCs. The percentage of fluorescenated cells in these cultures was similar to cultures that contained FITC-VLPs alone (Figure 1C).

To determine the magnitude of VLP cell uptake, intracellular fluorescence was measured by quenching extracellular fluorescence after exposure of cells to trypan blue prior to flow cytometric analysis [27]. Although the resulting fluorescence intensities of DCs incubated with FITC-VLPs were now lower following this treatment, the levels were still notably higher than untreated cells confirming the presence of intracellular FITC-VLPs (Figure 1D). Equivalent fluorescence cell intensities were also observed in DCs that were incubated with FITC-VLPs pre-mixed with E_8_Pam_2_Cys. However, a higher percentage of fluorescenated cells were detected in those cultures compared to those exposed to FITC-VLPs alone (Figure 1E) indicating that an increase in uptake of these constructs is facilitated using the lipopeptide.

**Figure 2:**
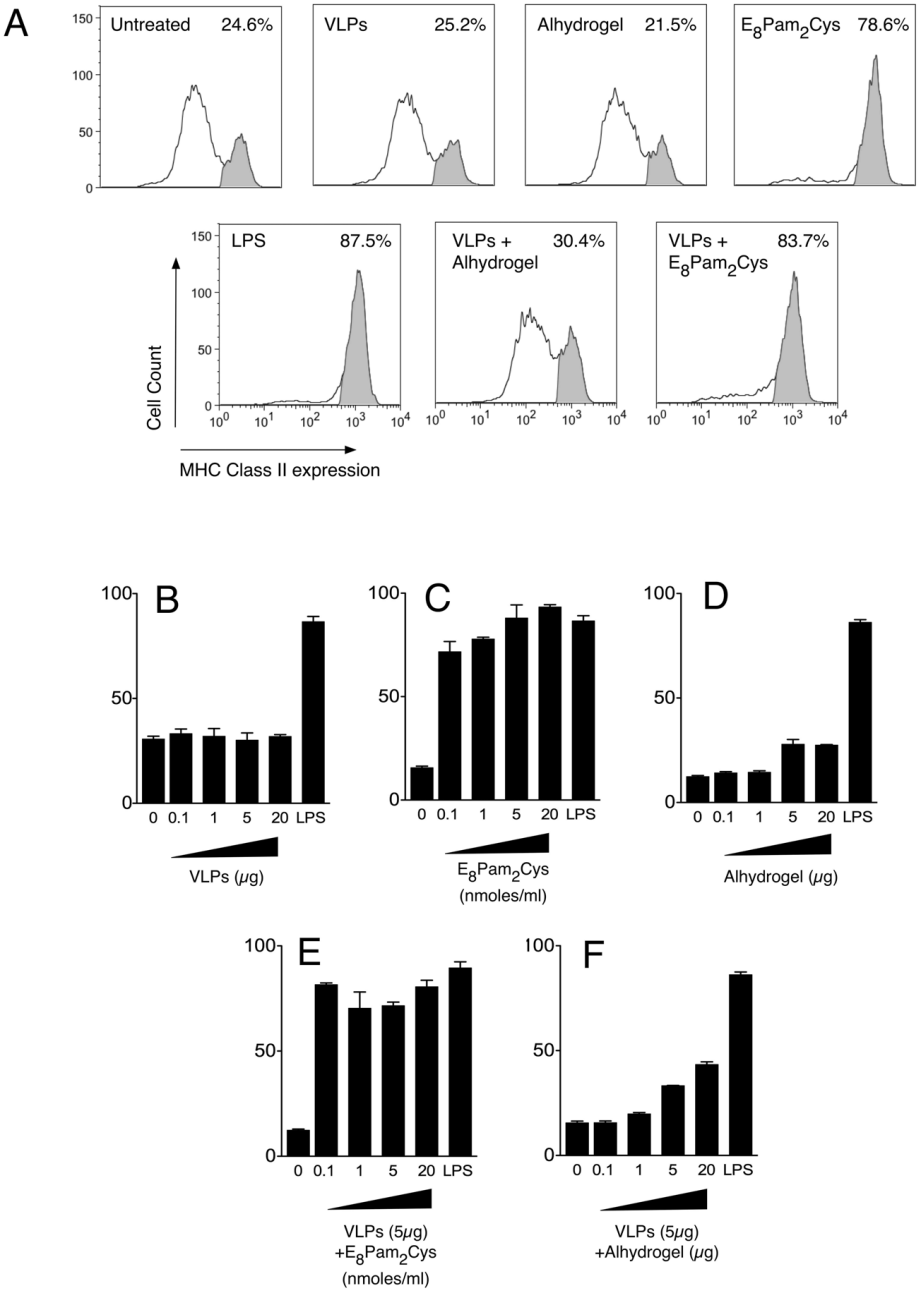
Dendritic cell maturation. (A) D1 dendritic cells (2×10^5^ cells) were incubated with VLPs (5 µg) alone or formulated with E_8_Pam_2_Cys (0.01 nmoles/ml) or Alhydrogel (5 µg) in a total volume of 1 ml. For comparative purposes within all experiments, cells were also either left untreated, exposed to LPS (5 µg/ml) or to similar amounts of each adjuvant alone. Cell surface MHC class II expression was determined after 16 hours using a PE-conjugated anti-IA/IE antibody. Cells expressing low levels of MHC Class II molecules were deemed to be immature whilst those expressing high levels were considered to be mature. Shown are representative histograms depicting cell surface MHC class II expression from one of three experiments conducted separately. MHC Class II^high^ expressing cells are shaded in grey. For dosing experiments, cells were also incubated with increasing amounts of (B) VLPs, (C) E_8_Pam_2_Cys, (D) Alhydrogel or VLPs (5 µg) formulated with increasing amounts of (E) E_8_Pam_2_Cys, or (F) Alhydrogel.

To investigate the ability of HCV VLPs and E_8_Pam_2_Cys to cause activation of DCs, we measured the expression of surface MHC class II molecules following incubation with the various antigens. The results (Figure 2A) indicate that untreated DCs contained two populations of cells which were MHC class II^low^ and MHC class II^high^, the latter comprising ∼24% of the population analysed. While the distribution of these populations was not affected by exposure to HCV VLPs alone, incubation with HCV VLPs mixed with E_8_Pam_2_Cys caused a dramatic shift in the distribution of MHC class II expressing cells such that 83% of cells were MHC class II^high^. The upregulation of MHC class II expression on these cells were comparable to those cultured in the presence of LPS which is a potent DC maturation stimulus.

Further dosing experiments showed that increasing the concentrations of HCV VLPs to 20 µg/ml, did not induce DC activation because the percentage of MHC Class II^high^ DCs in cultures containing escalating doses of VLPs remained similar to those containing untreated DCs (Figure 2B). In contrast, exposure to as little as 0.1 nmoles of E_8_Pam_2_Cys was sufficient at inducing a greater than two-fold increase in activation of DC compared to untreated cells (Figure 2C) and was similar to the levels of activation observed with LPS. No DC activation was observed in the presence of Alhydrogel (Figure 2D). The presence of E_8_Pam_2_Cys in HCV VLP-containing formulations however, not only promotes uptake of HCV VLPs by DCs but also considerably increases the level of DC activation.

### Enhanced HCV VLP-specific Antibody Responses Induced by Formulation with E_8_Pam_2_Cys

To determine if the DC activating properties of VLP formulations containing E_8_Pam_2_Cys translate to an improvement in HCV VLP immunogenicity, BALB/c mice were inoculated with VLPs alone or with VLPs mixed with E_8_Pam_2_Cys. HCV VLP-specific antibody titres in sera obtained after one, two or three doses of each formulation were then determined by ELISA.

**Figure 3:**
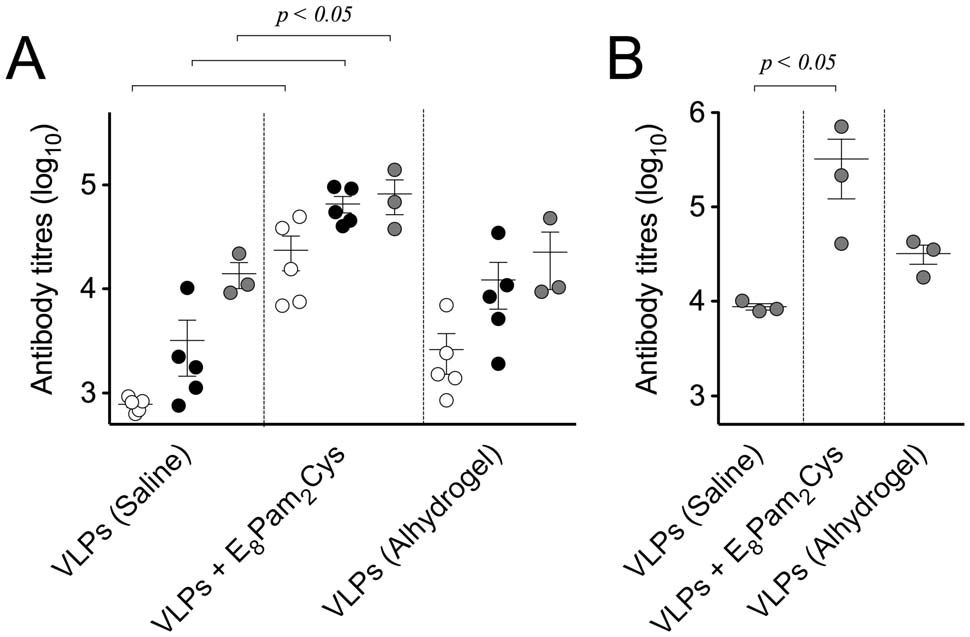
Antibody responses elicited by vaccination. Groups of BALB/c mice (n = 5 per group) were inoculated (100 µl) sub-cutaneously at the base of the tail with 20 µg of VLPs alone, pre-incubated with an equal volume of aluminium hydroxide (13 mg/ml) or 60 µg of E_8_Pam_2_Cys in saline on days 0 and 21. (A) VLP-specific antibody levels in sera prepared from blood taken on days 21 (white circles) and 35 (black circles) were then determined by ELISA. Individual animal antibody titres are presented with the mean value represented by the horizontal bar. An additional dose of each formulation was also administered to 3 mice from each group on day 56 and antibody titres determined on day 63 (grey circles). (B) E2-specific antibody titres in these animals were also determined at this same time point.

Administration of VLPs alone in saline was able to elicit detectable titres of specific antibody that were marginally increased after each dose of antigen (Figure 3A). In animals that received HCV VLPs mixed with E_8_Pam_2_Cys, however, antibody levels were significantly higher, in some cases by up to ten-fold more than those from animals that received the same dose of HCV VLPs alone. In fact the titre of specific antibody induced following administration of 3 doses of HCV VLPs alone was achieved using a single dose only of HCV VLP mixed with E_8_Pam_2_Cys. When compared to animals that were inoculated with HCV VLPs formulated with Alhydrogel, an adjuvant widely used to induce antibody responses to both human and veterinary vaccines [29], lower antibody titres were observed in these animals than in those that received the VLP-lipopeptide formulation. In examining levels of E2 specific antibodies elicited by vaccination, higher titres were once again demonstrated in animals that received 3 doses of VLPs mixed with E_8_Pam_2_Cys compared to those that were inoculated with VLPs alone or with Alhydrogel (Figure 3B).

**Figure 4:**
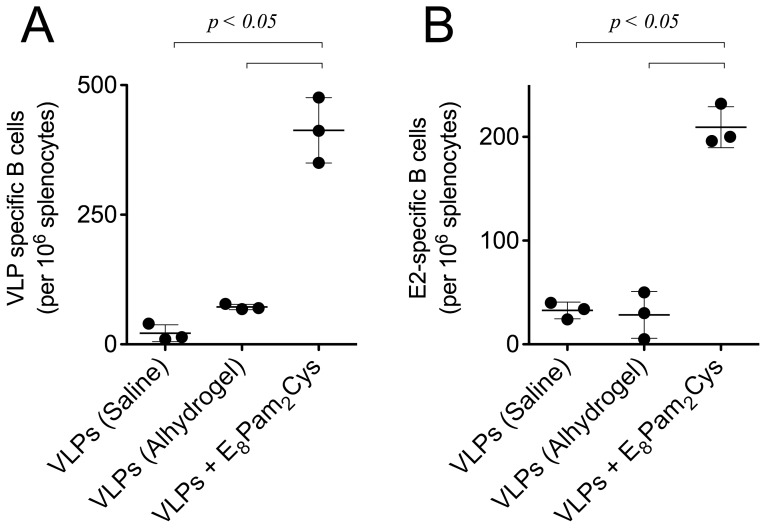
Induction of antibody secreting cells. BALB/c mice (n = 3) were inoculated with (100 µl) subcutaneously at the base of the tail with 20 µg of VLPs alone, pre-incubated with an equal volume of aluminium hydroxide (13 mg/ml) or 60 µg of E_8_Pam_2_Cys in saline on days 0, 21 and 56. Splenocytes were harvested on day 63 and frequencies of (A) VLP- or (B) E2-specific antibody secreting cells were enumerated in a B cell ELISPOT assay. Spot forming units representing the number of specific antibody-secreting cells in each animal is shown with horizontal bar indicating the average number of cells and standard deviation within each group.

The hierarchical pattern of antibody responses induced by E_8_Pam_2_Cys and Alhydrogel was also confirmed by the numbers of specific antibody secreting cells that were detected in the spleens of vaccinated mice. Once again significantly higher numbers of cells secreting both HCV VLP (Figure 4A) or E2-specific antibodies (Figure 4B) were detected in animals that received HCV VLPs mixed with E_8_Pam_2_Cys than those that were inoculated with HCV VLPs alone or VLPs formulated with Alhydrogel.

### Ability of Sera from Vaccinated Animals to Neutralise VLP Cell Entry

**Figure 5:**
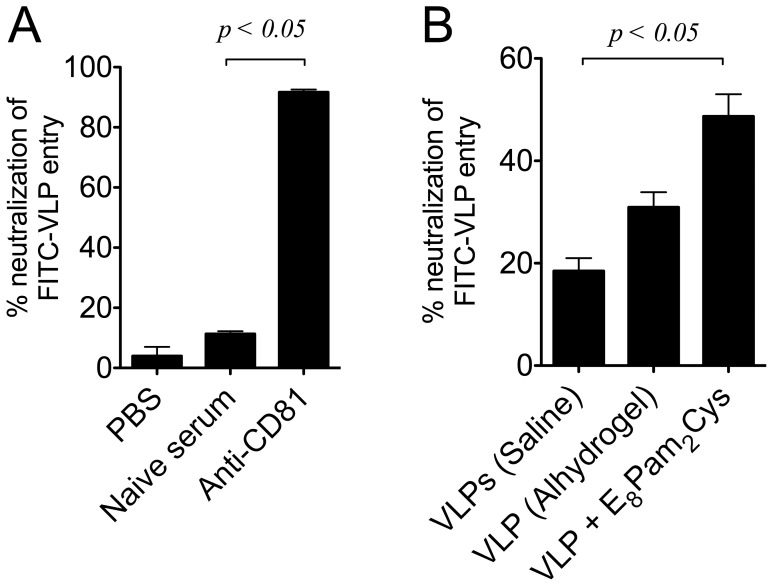
Neutralisation of VLP cell entry by sera from vaccinated animals. (A) Huh7 cells (5×10^5^ cells) were incubated with FITC labelled VLPs (200 ng) in the presence of a 1∶40 dilution of anti-CD81 antibody or serum from naïve mice. Cells were then washed and analysed for fluorescence by flow cytometry. (B) Neutralisation of VLP entry was determined by pre-incubating FITC labelled VLPs (200 ng) with a 1∶5 dilution of immune sera from mice inoculated with VLPs in saline, Alhydrogel or E_8_Pam_2_Cys. Supernatants were clarified by centrifugation, incubated with Huh7 cells (5×10^5^) in a total volume of (500 µl) for 1 hour. Cells were then harvested and cellular fluorescence levels analysed by flow cytometry. All bar graphs represent the percentage reduction in VLP entry relative to baseline levels obtained using serum from naïve mice.

To assess the neutralising activity of antibodies induced by vaccination, we first set out to investigate if VLP entry into human hepatocyte cell line Huh7 could be inhibited. Pre-incubation of FITC-labelled VLPs (VLP-FITC) with PBS or naïve serum resulted in minimal inhibition of VLP entry (Figure 5A). However, the presence of an antibody against CD81, a cell surface molecule implicated in HCV entry into hepatocytes [30], was able to prevent VLP entry into these cells by >90% confirming that these VLPs also utilise this molecule to facilitate cell entry. We next analysed the ability of sera obtained from mice inoculated with VLPs to inhibit the binding and entry of VLPs into Huh 7 cells (Figure 5B). Neutralisation of binding of VLPs to Huh7 cells was significantly greater in sera obtained from mice inoculated with VLPs in E_8_Pam_2_Cys (∼50%) compared to sera obtained from mice inoculated with VLPs administered in Alhydrogel (∼30%) or in saline (∼20%).

### Enhanced VLP-specific Cell-mediated Responses Induced by Formulation with E_8_Pam_2_Cys

**Figure 6:**
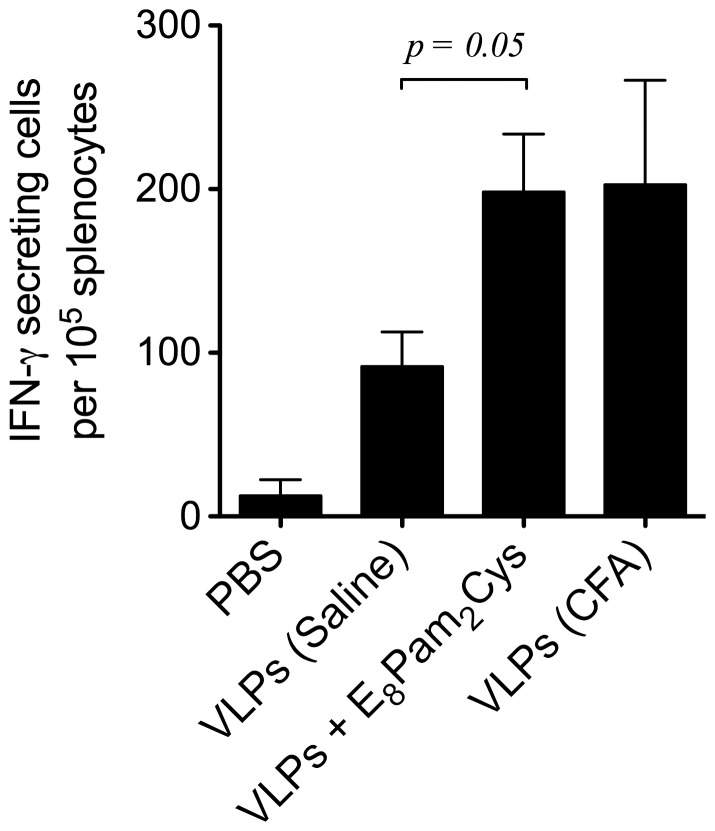
Cell-mediated responses elicited by vaccination. HLA-A2k^b^ transgenic mice (n = 3 per group) were inoculated (100 µl) subcutaneously at the base of the tail on days 0 and 14 with 30 µg of VLPs alone, emulsified with an equal amount of complete freund’s adjuvant (CFA) or pre-mixed with 30 µg of E_8_Pam_2_Cys in saline. Splenocytes were obtained 28 days later and restimulated for 7 days in the presence of 10 µg VLPs or an irrelevant HCV-derived HLA-A2-restricted epitope not part of the VLP construct. The frequency of peptide-specific T cells producing IFN-γ was determined in an ELISPOT assay. Each bar represents the average number of IFN-γ producing T cells and standard deviation in each group after subtracting non-specific responses from corresponding samples stimulated with the irrelevant peptide.

The ability of VLP formulations containing E_8_Pam_2_Cys to induce a cell-meditated immune response was examined by inoculating transgenic mice expressing the MHC class I (HLA-A2) allele but not endogenous H-2D^b^ molecules [28]. Control transgenic animals were inoculated with VLPs alone or VLPs emulsified with an equal amount of complete Freund’s adjuvant (CFA). Splenocytes from vaccinated animals were obtained 28 days post-inoculation and re-stimulated with antigen *in vitro*. The results (Figure 6) of an ELISPOT assay carried out revealed significantly higher numbers of HCV VLP-specific IFN-γ producing cells in the spleens of mice inoculated with VLPs in the presence of E_8_Pam_2_Cys or VLPs emulsified in CFA than in those that received VLPs alone.

## Discussion

The development of novel, effective anti-viral vaccine strategies in recent times has seen a notable shift away from the use of traditional formulations which utilize whole inactivated or live attenuated viruses towards approaches based on recombinant subunit protein antigens which are more easily characterised and defined. VLPs offer features that make them a useful platform for delivering viral antigens in a single vaccine construct which not only minimises the risks that may be associated with preparations containing or requiring the use of a replicating pathogen but will also closely resemble native viral antigens from which they are derived. The most convincing demonstration of VLPs efficacy is the quadrivalent VLP-based vaccine Gardasil which prevents persistent infection and associated disease caused by human papillomavirus [31]. Other studies of VLP-based vaccination strategies have also shown promising results and led to Phase I testing against a number of disease indications including seasonal and pandemic influenza, Hepatitis B, malaria and HIV (reviewed in [32]).

Depending on the type of virus used to manufacture a VLP construct, studies have shown that protective responses induced by VLPs can be elicited without co-administration of adjuvant [33]–[35]. In many cases, however, the induction of useful immune responses may require multiple doses [36], [37], a regime that may be impractical to implement in the field or involve a viral vector to provide an initial priming dose followed by a boost using VLPs [22], [38]. Of relevance to the present study, the use of adjuvants to enhance VLP immunogenicity has been shown to induce strong antibody responses using dose-sparing amounts of HIV [39] or Norwalk virus-derived VLPs [40] and also elicits cell-mediated responses that culminate in improved protection against lethal influenza viral [41], [42] and tumorigenic challenge [43].

Our previous studies have shown peptide epitope and protein-based antigens can be made far more immunogenic when covalently attached to Pam_2_Cys in order to target their delivery via TLR2 to dendritic cells (DCs) [44]. This results in the induction of robust antibody and CD8^+^ T cell-mediated immune responses and has been shown for multiple indications [44]–[47]. Each of these vaccine candidates demonstrated the ability of this simple lipid structure to dramatically enhance the immunogenicity of antigens that are otherwise immunologically inert. Nevertheless, the approach introduces complexities into the vaccine manufacturing process due to the requirement for covalent attachment of Pam_2_Cys to an antigen. The use of the anionic lipopeptide E_8_Pam_2_Cys overcomes many of the technical complexities related to this process, especially the use of covalent chemistries, by making use of electrostatic association with antigen [23].

The ability of Pam2Cys to dramatically enhance the immunogenicity of HCV VLPs was demonstrated in the improved overall antibody responses that we observed. Not only are greater antibody titres induced following vaccination with VLPs formulated with E_8_Pam_2_Cys compared to the use of VLPs alone or when co-administered with Alhydrogel but up to 3 doses of non-adjuvanted or traditionally adjuvanted antigen were required to match the titres obtained with a single dose using lipopeptide. Most importantly in the context of HCV the trend translates to improved E2-specific antibody responses and the use of lower doses of VLPs to achieve this while maintaining efficacy has major advantages by providing cost benefits to vaccine manufacturers.

Our studies examining the interaction of VLPs with DCs indicate that while improvements in VLP uptake mediated by this lipopeptide is minimal, its ability to concomitantly induce the maturation of DCs at very small doses is a feature not observed using VLPs alone or VLPs administered in the presence of alum. Our previous work also demonstrated that association of antigen with charged lipopeptide facilitates trafficking of antigen to lymph nodes draining from the vaccination site [23] and together with the results presented in this study provide an explanation for the dose-sparing neutralising antibody responses that we observe. Co-administration of VLPs using charged lipopeptide has the added benefit of eliciting VLP-specific cell-mediated responses in HLA-A2 transgenic mice, a fact that may also be attributed to DC targeting and activation. Given the results of the work described in this study, we conclude that the use of this branched anionic lipopeptide together with VLPs containing HCV antigens in order to provide a broad spectrum of conformational epitopes can provide benefits in terms of inducing improved levels of neutralising antibody titres and eliciting cell-mediated responses. This strategy could therefore constitute a valuable addition to the armamentarium of current VLP-based vaccine developments against HCV.
